# Dual-color core–shell silica nanosystems for advanced super-resolution biomedical imaging†

**DOI:** 10.1039/d3na00310h

**Published:** 2023-08-25

**Authors:** Maria Antonieta Ramirez-Morales, Elisa De Luca, Chiara Coricciati, Alberto Rainer, Giuseppe Gigli, Giuseppe Mele, Pier Paolo Pompa, Maria Ada Malvindi

**Affiliations:** a HiQ-Nano s.r.l. Via Barsanti 1, Arnesano Lecce 73010 Italy maryarmzm@gmail.com mariada.malvindi@hiqnano.com; b Department of Engineering of Innovation, Università del Salento Via Monteroni Lecce 73100 Italy; c Institute of Nanotechnology (NANOTEC)-National Research Council (CNR) Lecce 73100 Italy; d Center for Biomolecular Nanotechnology (CBN), Istituto Italiano di Tecnologia Via Eugenio Barsanti, 1, Arnesano 73010 Italy; e Department of Mathematics and Physics “Ennio De Giorgi”, University of Salento Lecce 73100 Italy; f Università Campus Bio-Medico di Roma Via Álvaro del Portillo 21 Roma 00128 Italy; g Nanobiointeractions & Nanodiagnostics, Istituto Italiano di Tecnologia (IIT) Via Morego 30 Genova 16163 Italy

## Abstract

Fluorescent core–shell silica nanoparticles are largely employed in nanomedicine and life science thanks to the many advantages they offer. Among these, the enhancement of the stability of the fluorescent signal upon fluorophore encapsulation into the silica matrix and the possibility to combine in a single vehicle multiple functionalities, physically separated in different compartments. In this work, we present a new approach to the Stöber method as a two-cycle protocol for the tailored synthesis of dual-color fluorescent core–shell silicon dioxide nanoparticles (SiO_2_ NPs) using two commercial dyes as model. To facilitate the colloidal stability, the nanoparticle surface was functionalized with biotin by two approaches. The biotinylated nanosystems were characterized by several analytical and advanced microscopy techniques including Fourier transform infrared (FT-IR) spectroscopy, dynamic light scattering (DLS), UV-vis, transmission electron microscopy (TEM) and confocal laser scanning microscopy (CLSM). Moreover, advanced super-resolution based on structured illumination was used for the imaging of the double-fluorescent NPs, both on a substrate and in the cellular microenvironment, at nanometric resolution 100 nm, in view of their versatile potential employment in fluorescence optical nanoscopy as nanoscale calibration tools as well as in biomedical applications as biocompatible nanosystems for intracellular biosensing with high flexibility of use, being these nanoplatforms adaptable to the encapsulation of any couple of dyes with the desired function.

## Abstract 2

Tailored synthesis and biotin-functionalization of dual-colored SiO_2_ nanoparticles as tools for advanced super-resolution microscopy imagining in biomedical approaches.

## Graphical abstract

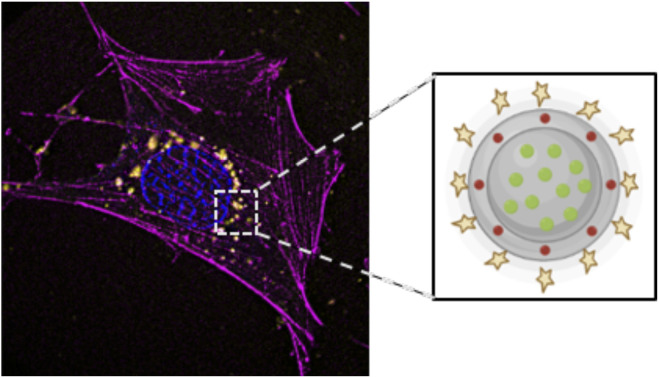

## Introduction

In recent years, multiple nanoparticle-based tools have been developed for biomedical applications drug delivery,^1,2^ antibacterial activity,^3,4^ sensing,^5–8^ cancer therapy,^9^ imaging and tissue engineering.^10–12^ Focusing on imaging, dye-loaded SiO_2_ NPs^11–13^ are among the most used nanomaterials due to their advantageous properties, such as biocompatibility,^14,15^ tunable size, good morphology and dispersion, and easy surface chemistry modification which allow molecules to be chemically bonded to their surface.^13,16,17^ SiO_2_ NPs are generally synthesized *via* hydrolysis and condensation of tetraethyl orthosilicate (TEOS)^17–20^ obtaining a wide size range from 5 to 2000 nm.^19^ Although several methods like microwaving and chemical vapour condensation (CVC) have been attempted,^19,20^ the sol–gel process (Stöber) and reverse microemulsion are still the most used techniques.^17–22^

Multiple-colored fluorescent NPs can optimize diagnostic imaging and therapeutic techniques by providing accurate control and monitoring of the interactions inside living cells between the target organelles and the used nanomaterial.^1,23–25^ As reported in the literature, several fluorescent molecules have been attached on NPs surface^26,27^ and different colours of fluorescent SiO_2_ NPs have been developed. SiO_2_ NPs have been functionalized with rhodamine,^6^ cyanine,^28^ have been doped with Cd/RhBITC,^29^ Oregon Green 488 (OG 488),^30^ fluorescein isothiocyanate (FITC),^31^ ATTO 647N, STAR 635, Dy-647, Dy-648 and Dy-649.^32,33^ Multicolor systems, with a size above 100 nm, has been also reported as calibration tools for imaging instruments^34,35^ for the precise and accurate analysis in life science and biomedicine studies.^36^

Despite the multiple advantages of fluorescent silica systems, SiO_2_ NPs tend to agglomerate in solution, especially in cell culture media,^34^ limiting their studies in biological systems. In order to increase the colloidal stability of NPs, different surfactants/ligands^17,18,35–37^ can be added to the solution^38,39^ using two common approaches: surface adsorption and covalent attachment.^14^ In this work, we adsorbed biotin on NPs surface to improve their dispersion in solution and to increase stability. Moreover, biotin could be used for further studies to improve the nanoparticles uptake in cancer cells,^43–45^ due to overexpression of biotin receptors in tumoral cells.^40–42^ Here, we propose a tailored synthesis to obtain well-defined dual-color emitting NPs, with the potential use in the biomedical field as bioimaging/sensing tool.

## Results and discussion

**Fig. 1:**
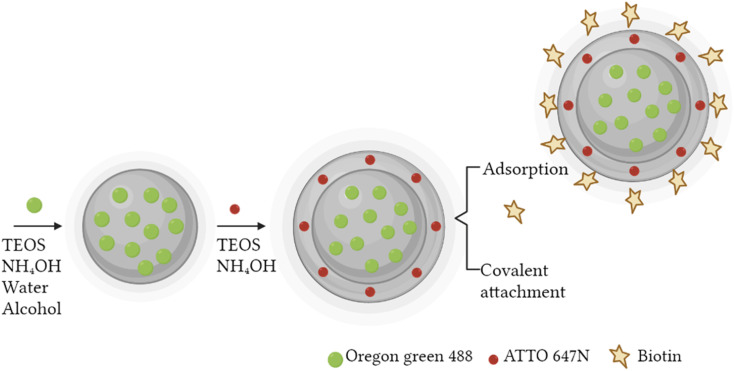
Schematic representation of two-cycle Stöber method (created by Biorender).

A fast, simple, and reproducible method for the synthesis of dual-color SiO_2_ NPs is proposed herein. The two-cycle Stöber method (Fig. 1) is based on the sol–gel process creating two loaded silica matrices with a fluorescent dye in each. First, a silica core incorporating a covalently bond green fluorophore was created. Subsequently, the second layer of fluorescent silica (shell) was formed through a second process of silica growing and far-red dye loading. Those nanosystems can be imaged using different excitation wavelengths and different emission ranges, to get a better understanding of the behaviour of NPs in complex cellular systems, such as internalization, release, degradation, distribution. Upon optimization, such a robust method allowed us to achieve fine control and tunability of the particle size.

**Fig. 2:**
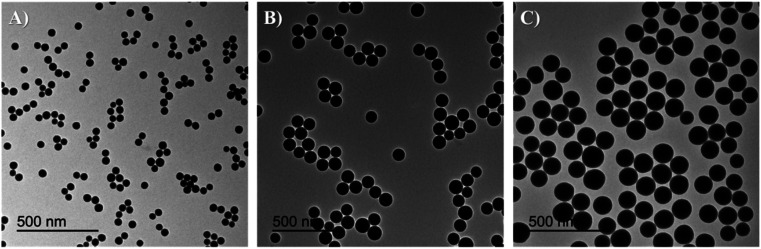
TEM images and size distribution of core–shell nanosystems (A) DC 50 (49 ± 3 nm), (B) DC 90 (93 ± 4 nm) and (C) DC 120 (116 ± 3 nm).

**Fig. 3:**
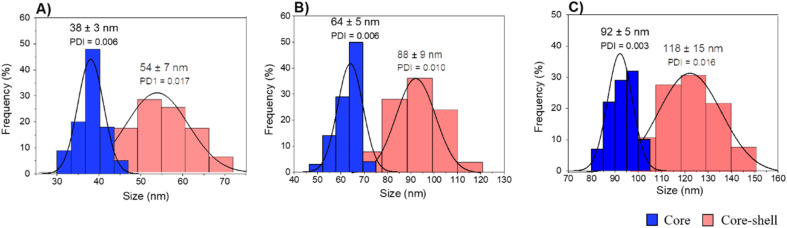
DLS measurements of core and core–shell silica-based nanosystems in water (A) DC 50, (B) DC 90 and (C) DC 120.

TEM images of the different core–shell particles (Fig. 2) showed well monodispersed and spherical shaped nanosystems with sizes of 49 ± 3 nm (DC 50), 93 ± 4 nm (DC 90) and 116 ± 3 nm (DC 120). Their size distribution was evaluated also in water solution through DLS measurements. As showed in Fig. 3, at the end of the first cycle NPs had a size respectively of 38 ± 3 nm, 64 ± 5 nm and 92 ± 5 nm, and a final core–shell nanoparticle size of 54 ± 7 nm, 88 ± 9 nm and 118 ± 15 nm.

**Fig. 4:**
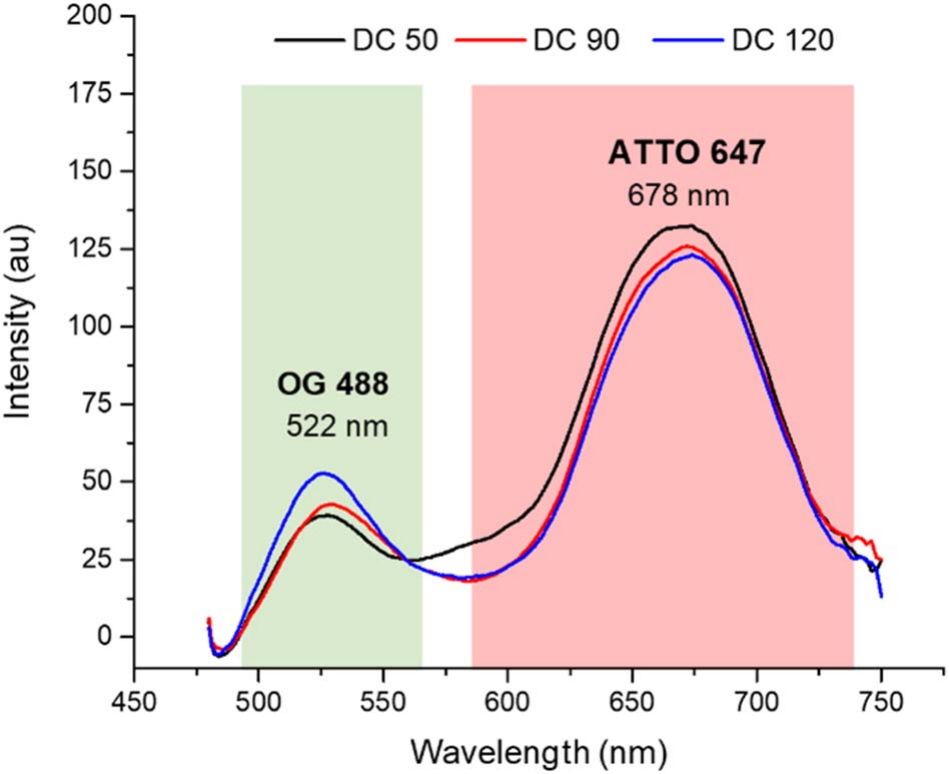
Normalized fluorescence spectra of DC 50, DC 90 and DC 120 in ethanolic solution (*λ*_ex_ = 450 nm).

After the morphological characterization, the fluorescence emission spectra (Fig. 4) were measured for both dyes in a wavelength range from 470 to 750 nm. Although the concentrations of the two dyes in solution were equivalent (confirmed by calibration curves in ESI S1†), comparing the emission spectrum of dual color NPs with that of single color NPs (in ESI S2a†) we observed a decreased emission of OG 488 and an increasing in the emission of ATTO 647N. The lower fluorescence intensity of OG 488 can be attributed to different factors. First, a greater amount of ATTO 647N is encapsulated in the shell, compared to the amount of OG 488 encapsulated in the core, since the shell had a significantly greater volume than the core. Second, it may be due to a partial Förster resonance energy transfer (FRET) phenomenon. According to the literature, for an efficient FRET to occur, the maximum distance between the fluorescent donor and its corresponding acceptor must be less than 10 nm.^43–45^ In our nanosystems not all fluorophores are within the FRET distance optimal limits, leading to a partial FRET process. In addition, there may be a small contribution of the silica matrix that adsorbs some of the green fluorescence. As confirmed by the spectra in ESI S3,† when the dyes are dispersed in solution, there is not a significant fluorescent emission signal decrease.

To improve the dispersion and stability of the colloidal suspensions a biotin surface functionalization step was performed. Biotin not only permits to decrease nanoparticle agglomeration, but also to enhance the cellular uptake,^46,47^ thanks to the increase of nanoparticle tissue specificity and to the overexpression of biotin receptors on the cell membrane, mainly present in breast cancer and in others types such as colon, leukaemia and lung cancer.^48^ Both biotin and biotin–NHS were tested for the nanosystem functionalization using three different mass ratio between the biomolecule and the nanosystem DC 50 (1 : 1, 1 : 3, 1 : 5). Zeta potential measurements were carried out (Table 1) to confirm the successful functionalization and evaluate the optimal conditions of dispersion and stability of the NP suspensions. Pristine dual-colored SiO_2_ NPs used as control showed a negative potential value 39.76 ± 0.47 mV, expected by the negative charges on the surface due to OH groups. The biotin adsorption on the NPs surface led to an important change of the zeta potential to 60.6 ± 2 mV in the ratio 1 : 1. The increase of biotin amount (ratio 1 : 3) did not show a significant change of zeta potential (62.7 ± 1 mV), suggesting biotin saturation of the NPs surface. Similar results were obtained using biotin–NHS. The ratio 1 : 1 (biotin : NPs) confirmed the best results, with a zeta potential of 62.8 ± 1 mV, since by increasing the amount of NPs, the potential change decreases considerably. We considered optimal the zeta potential obtained after the adsorption procedure with the ratio 1 : 1 (biotin : NPs) and decided to use it in our next experiments, since no additional steps are required for the functionalization, it was a faster and cheaper procedure then the carboxylic acid group activation using biotin–NHS. The presence of biotin on the NPs surface was confirmed using the commercial HABA/Avidin kit (SIGMA H-2153) and through FT-IR measurements. The concentration of biotin measured with HABA/Avidin kit was close to 3 mg mL^−1^, confirming the amount added for the conjugation (ESI S4†). The presence of biotin on DC 50 surface was also confirmed by the reported signals of both silica and biotin (ESI S5†).

**Table 1:** Zeta potential of suspensions using different mass ratio between biotin and nanoparticles^a^

Approach	Mass ratio biotin : NPs	*Z*-Potential (mV)
Biotin adsorption	1 : 1	60.6 ± 2
	1 : 3	62.7 ± 1
	1 : 5	14.7 ± 2
Covalent linking	1 : 1	62.8 ± 1
	1 : 3	21.8 ± 2
	1 : 5	10.3 ± 1

a Control solution: (−) 39.8 ± 0.5 mV.

**Fig. 5:**
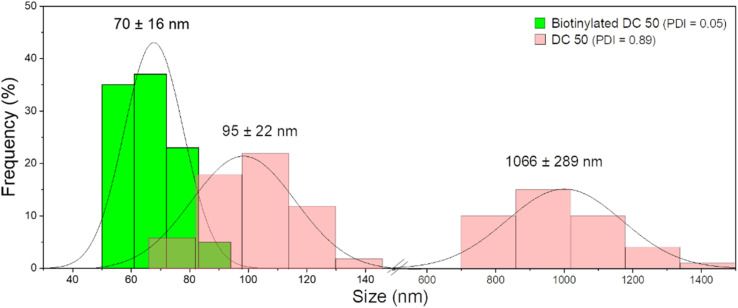
DLS measurements of DC 50 in cell culture medium (DMEM) before and after biotinylation.

After functionalization of DC 50 NPs, their dispersion in cell culture medium was evaluated, comparing their size distribution with pristine NPs without biotin on the surface. As confirmed by DLS measurements (Fig. 5), the NPs without biotin tended to form large aggregates in cell culture medium observed by two groups with average diameter of 95 ± 22 nm and 1066 ± 289 nm having a PDI equals to 0.89, while the biotin on the NP surface leads to the formation of protein corona but significantly reduced the particle aggregation up to 70 ± 16 nm, increasing the stability and dispersion (PDI = 0.05).

**Fig. 6:**
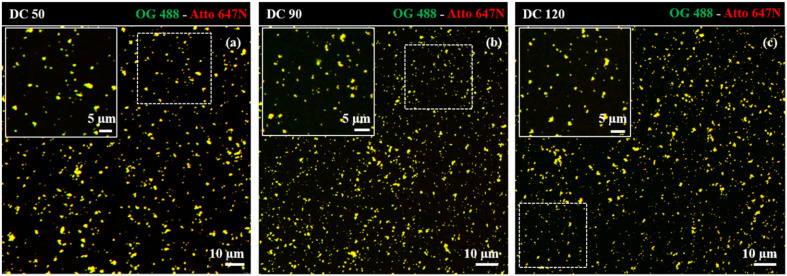
Confocal imaging of dual-color SiO_2_ NPs (a) DC 50, (b) DC 90 and (c) DC 120 immobilized on coverslip. Insets display close-up views (zoom-in) of the regions marked by the dotted white square.

Dual-color biotin-functionalized NPs were imaged by CLSM. As shown in Fig. 6, dyes-loaded SiO_2_ NPs show a good dispersion on the substrate for all the sizes, and a high fluorescence brightness of both fluorescent probes. However, due to the diffraction-limited nature of confocal imaging^49,50^ (lateral resolution of approximately 200–250 nm) we pushed the imaging of NPs to the nanoscale, adopting structured illumination microscopy (SIM), a super-resolution approach that provides an improvement in spatial resolution of a factor of two when compared to conventional fluorescence microscopy.^51^ This optical nanoscopy technique has the great advantage to exploit the high frame rate acquisition and the relatively low illumination light power to reconstruct the image of the illuminated object using a conventional wide-field microscope and standard sample preparation protocols. To further improve the quality of the imaging and the achievable resolution for NPs characterization, we employed an evolved version of SIM technology, namely Lattice SIM^2^, which benefits from the combination of optical 3D lattice illumination patterns and the dual iterative reconstruction algorithm (Lattice SIM^2^), increasing signal to noise ratio and resolving structures below 100 nm in *x* and *y*, achieving an effective lateral resolution down to ∼60 nm.^52,53^

**Fig. 7:**
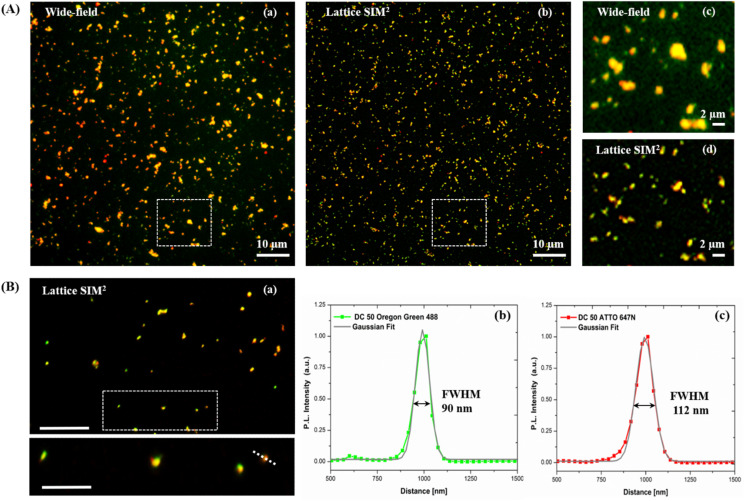
(A) Comparison between conventional diffraction-limited wide-field and super-resolution Lattice SIM^2^ images of dual-color SiO_2_ NPs DC 50. (a) Wide-field and (b) corresponding Lattice SIM^2^ image of the same field of view; (c) and (d) close-up view of the regions marked by the dotted white squares, showing the enhanced resolution of Lattice SIM^2^. (B) (a) Super-resolution Lattice SIM^2^ image (top image) and inset (bottom image) displaying a close-up view of the region marked by the dotted white square. Representative Gaussian fits (grey lines) of normalized intensity profiles of Oregon Green 488 (green curve) (b) and ATTO 647N (red curve) (c), along the dotted line in the inset of image (a). The FWHM values extracted from the Gaussian fit show the resolution enhancement attained with Lattice SIM^2^ imaging.

As shown in Fig. 7 panel (A), with SIM^2^ processing we achieved a significantly improved image contrast and lateral resolution compared to unprocessed imaging. Indeed, the FWHM values extracted from the Gaussian fit of Oregon Green 488 and ATTO 647N emission intensity profiles, relative to the fluorescent spot in the inset of panel (B), showed a remarkable improvement of the *xy* resolution down to *ca.* 90 nm, close to the maximum resolution limit achievable with this technique.

It is worth underlining that the core–shell configuration of the proposed NPs paves the way to the development of theragnostic tools, where stimuli responsive features of the dyes associated to the core and the shell of the nanoparticles could be used to develop nano-sized sensors. As a proof of principle, we leveraged the reported the peculiar feature of Oregon Green 488 dye, which is marketed as insensitive to pH changes in the near-neutral pH range but pH sensitive in moderately acidic solutions, thus displaying pH-emission dependency.^54^ We then measured the ratio between Green 488 and ATTO 647N emission intensities in DC 50 NP exposed to different pH values in the 3–14 range. We report a response of the nanoparticles to acidic pH levels, associated to a decrease in the ratiometric readout (see ESI S6†).

**Fig. 8:**
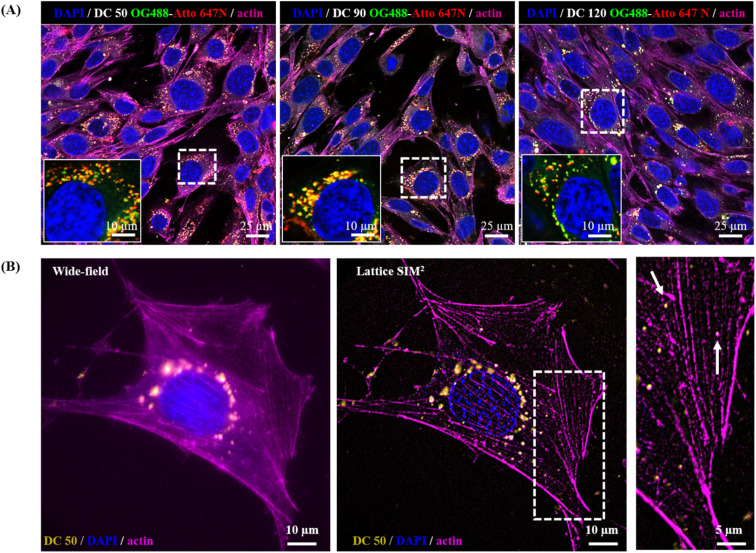
(A) NPs internalization in MCF7 cells. Representative confocal images of cells incubated for 24 hours with SiO_2_ NPs of different sizes (1 nM). Images display a large field of view with several cells; insets show the zoom-in on the single cells marked by the dotted white squares. Blue: nuclei stained with DAPI; magenta: actin cytoskeleton stained with phalloidin 568; yellow: OG 488/ATTO 647N NPs. (B) Comparison between wide-field and Lattice SIM^2^ imaging of cells incubated with DC 50. The inset displays a close-up view of the area marked by the dotted white squares.

In view of a future employment of our NPs as theragnostic tools in nanobiomedicine and as probes for high-resolution imaging, we investigated their behaviour within the biological microenvironment. We further confirmed the excellent fluorescent properties of the NPs by visualizing them within cells upon internalization (Fig. 8A) and observed their prominent uptake (thanks to their good dispersion in culture medium) *via* endocytic pathway and endo-lysosomal accumulation at the proximity of the nucleus, in line with the precedent reported literature on silica NPs.^14,55,56^

The NP internalization was also followed by Lattice SIM^2^, to improve the quality of NP detection over confocal microscopy. The imaging of sub-diffraction nanostructures at a resolution close to their effective size is strongly recommended to fully explore their behaviour, particularly with respect to the investigation of nano–bio interactions at the molecular scale. Fig. 8, panel (B) highlights the enhancement of resolution attained with SIM^2^ processing that enables to distinguish small clusters/single NPs and sub-cellular structures at high contrast with nanoscale spatial resolution, paving the way for advanced multicolour dynamic imaging of nanomaterial–biosystem interactions, essential to improve their rational design for biomedical applications.

## Conclusions

In this work, we have developed an easy and fast two-cycle Stöber synthesis to prepare monodisperse and well-defined dual-color SiO_2_ NPs that can be used in biomedical applications. The core–shell NPs present two different commercial dyes (Oregon Green 488 and ATTO 647N) trapped in different silica matrices and a biotinylated surface, functional to optimize nanoparticle colloidal stability and to their perspective use in selective cell cancer targeting. Here, we characterize the dual emitting fluorescent nanosystem in view of its potential use in both optical nanoscopy imaging and nanobiosensing.

## Experimental

### Synthesis of dual-color nanosystems

A two-cycle Stöber method was used for the tailored synthesis of dual-colored core–shell SiO_2_ NPs. For the first cycle, Oregon Green 488-doped core was obtained mixing 20 μL of dye (previously activated with APTES), 880 μL of Triton X-100, 3.75 mL of cyclohexane, 900 μL of alcohol (1-octanol and 1-hexanol for 50 and 100 nm core respectively), 170 μL of Milli-Q water, 20 μL of TEOS and 120 μL of ammonium hydroxide (28%) by a vigorously stirring. Ethanol was added to stop the reaction after 24 hours. The suspensions were purified and fixed to a concentration of 0.1 mg μL^−1^.

For the second cycle, the ATTO 647N doped shell was obtained by stirring 1000 μL of the previous solution with 20 μL of TEOS, 900 μL of ammonium hydroxide (28%), 10 μL of dye (previously activated with APTES) and 5 mL of ethanol for another 24 hours at room temperature.

### Functionalization of dual-color nanosystems

All nanosystems were functionalized with biotin by two approaches: simple biotinylation and carboxylic acid groups (–COOH) activation using biotin–hydroxysuccinimide ester (NHS). Different mass ratios between the NPs suspensions and biotin were prepared (1 : 1, 3 : 1, and 5 : 1) by a bath sonicator for 30 minutes. The sonicated dispersions were purified and washed with ethanol three times by centrifugation at 14 500 rpm for 20 minutes, subsequently re-dispersed in water and stored at 4 °C.

### Characterization of dual-color nanosystems

DLS and zeta potential measurements were performed on a Zetasizer Nano ZS90 (Malvern, USA) equipped with a 4.0 mW HeNe laser operating at 633 nm and an avalanche photodiode detector. Measurements were made at 25 °C in aqueous solutions and in cell culture medium supplemented with 20% FBS at the final concentration 1 nM. Transmission electron microscope (TEM) images were recorded by a JEOL JEM 1011 microscope operating at an accelerating voltage of 100 kV. TEM samples were prepared by dropping a dilute solution of NPs in water on carbon-coated copper grids (Formvar/Carbon 300 mesh Cu). Fluorescent spectra were obtained in a 96-well plate with 20 μL of the sample diluted in 180 μL of ethanol. The fluorescence intensities were recorded from 470–750 nm using a TECAN 200 PRO. For acidic (pH 3.5) and basic (pH = 10 and 14) solutions, the pH was adjusted using hydrochloric acid (HCl) and sodium hydroxide (NaOH) respectively.

### Biotin determination

Spectrophotometric determination of biotin was measured using HABA/Avidin reagent (SIGMA H-2153) at 500 nm (ESI S4†). Fourier transform infrared (FT-IR) spectrum was measured at wavenumber from 500 to 4000 cm^−1^ using a JASCO FTIR-6800. Ethanolic suspensions were directly placed on the ray exposing stage.

### Cell cultures

MCF7 (human mammary gland adenocarcinoma cells ATCC HTB-22) were grown in DMEM medium (Sigma-Aldrich) supplemented with 10% (v/v) FBS (Sigma-Aldrich), 2 mM l-glutamine (Sigma-Aldrich), 100 U per mL penicillin and 100 mg per mL streptomycin (Sigma-Aldrich). The cells were incubated at 37 °C under a humidified controlled atmosphere with 5% CO_2_.

### Imaging

CLSM was performed using a Leica TCS SP8 confocal microscope (Leica Microsystems, GmbH, Germany) equipped with a 63× oil-immersion objective (HC PL APO CS2 63×/1.40 OIL, Leica Germany). Imaging performed using a white light laser (470–670 nm) and exciting OG 488 fluorophore at 488 nm, ATTO 647N fluorophore at 647 nm, DAPI (4′,6-diamidino-2-phenylindole) at 405 nm, and phalloidin 568 at 561 nm. Fluorescent emission was detected in the spectral window between 500 and 550 nm (OG 488 emission), 655–710 nm (ATTO 647N emission), 450–440 nm (DAPI emission), and 578–620 (phalloidin 568 emission) by a GaAsP hybrid photodetector (Leica HyD™).

Lattice SIM^2^ imaging was performed using Zeiss Elyra 7 with Lattice SIM and SMLM, using the SIM^2^ reconstruction algorithm. NPs were excited with the laser line at 488 nm (to excite OG 488 fluorophore), at 640 nm (to excite ATTO 647N fluorophore), and the emission signals have been detected with two synchronized cameras (Dual PCO.Edge 4.2 sCMOS camera with Duolink, motorized Dual Camera Adapter for two channel simultaneous acquisitions). DAPI was excited with a 405 nm laser line and phalloidin 568 with a 561 nm laser line and using the appropriate combinations of filters and beam splitters. All the data were acquired using ZEN black software (Zeiss) and analyzed using Origin Pro 8.5 software.

### Nanoparticles deposition for fluorescence optical imaging

An initial water suspension of 10 nM NPs was diluted (1 : 500) and drop casted on a coverslip (thickness of 0.17 mm); after evaporation, samples were covered by ProLong Gold Antifade Mountant (P36934, Thermo Fisher Scientific) as embedding medium in order to avoid mismatch of refractive index, and mounted on a glass microscope slide until the complete hardening of the mounting medium.

### Nanoparticles incubation for cellular imaging

MCF7 cells were seeded on μ-Slide 8 Well (80806, Ibidi) and incubated for 24 hours in a humified atmosphere at 37 °C and 5% CO_2_ to obtain a subconfluent monolayer (60–70% of confluence). After 24 hours the medium was removed, and the cells were incubated with a suspension of NPs (1 nM) for 24 hours of incubation at 37 °C, then cells were washed three times with PBS (pH 7.4) and stained with DAPI and Alexa Fluor 568 Phalloidin (A123-80, Thermo Fisher Scientific).

## Author contributions

Conceptualization, G. A. M., E. D. L., P. P. P., and M. A. M.; methodology, M. A. R. M., E. D. L., C. C. and M. A. M.; investigation, M. A. R. M. and E. D. L.; resources, E. D. L. and M. A. M.; writing-original draft preparation, M. A. R. M., E. D. L., and M. A. M.; writing, review and editing, G. A. M., P. P. P., A. R., G. G., and M. A. M.; supervision, G. A. M., P. P. P. and M. A. M.; project administration, M. A. M. All authors have read and agreed to the published version of the manuscript.

## Conflicts of interest

There are no conflicts to declare.

## Supplementary Material

NA-005-D3NA00310H-s001
