# Supplementary material for: Dual-color core–shell silica nanosystems for advanced super-resolution biomedical imaging
Source: Nanoscale Adv. 2023 Aug 25;5(21):5766–73. doi: 10.1039/d3na00310h (PMC10597559; doi:10.1039/d3na00310h)
Supplement: NA-005-D3NA00310H-s001 [file NA-005-D3NA00310H-s001.pdf]

Electronic Supplementary Material (ESI) for Nanoscale Advances.
This journal is © The Royal Society of Chemistry 2023

SUPPORTING INFORMATION

Dual-color core-shell silica nanosystems for advanced super-resolution biomedical imaging

Maria Antonieta Ramirez-Morales[1,2*†], Elisa De Luca[3,4†], Chiara Coricciati[3,5], Alberto Rainer[3,6], Giuseppe Gigli[3,5], Giuseppe Mele[2], Pier Paolo Pompa[7], and Maria Ada Malvindi[1*]

[1] HiQ-Nano s.r.l., Via Barsanti 1, 73010, Arnesano, Lecce, Italy
[2] Department of Engineering of Innovation, Università del Salento, Via Monteroni, 73100, Lecce, Italy
[3] Institute of Nanotechnology (NANOTEC)- National Research Council (CNR), 73100, Lecce, Italy
[4] Center for Biomolecular Nanotechnology (CBN), Istituto Italiano di Tecnologia, Via Eugenio Barsanti, 1, 73010, Arnesano, Italy
[5] Department of Mathematics and Physics "Ennio De Giorgi", University of Salento, 73100, Lecce, Italy
[6] Università Campus Bio-Medico di Roma, Via Álvaro del Portillo 21, 00128, Roma, Italy
[7] Nanobiointeractions & Nanodiagnostics, Istituto Italiano di Tecnologia (IIT), Via Morego 30, 16163, Genova, Italy

*Corresponding authors: maryarmzm@gmail.com, mariada.malvindi@hiqnano.com

†Maria Antonieta Ramirez-Morales and Elisa De Luca have equally contributed to this work

S1. Fluorescent emission and excitation spectra of Oregon Green 488 and ATTO 647N.

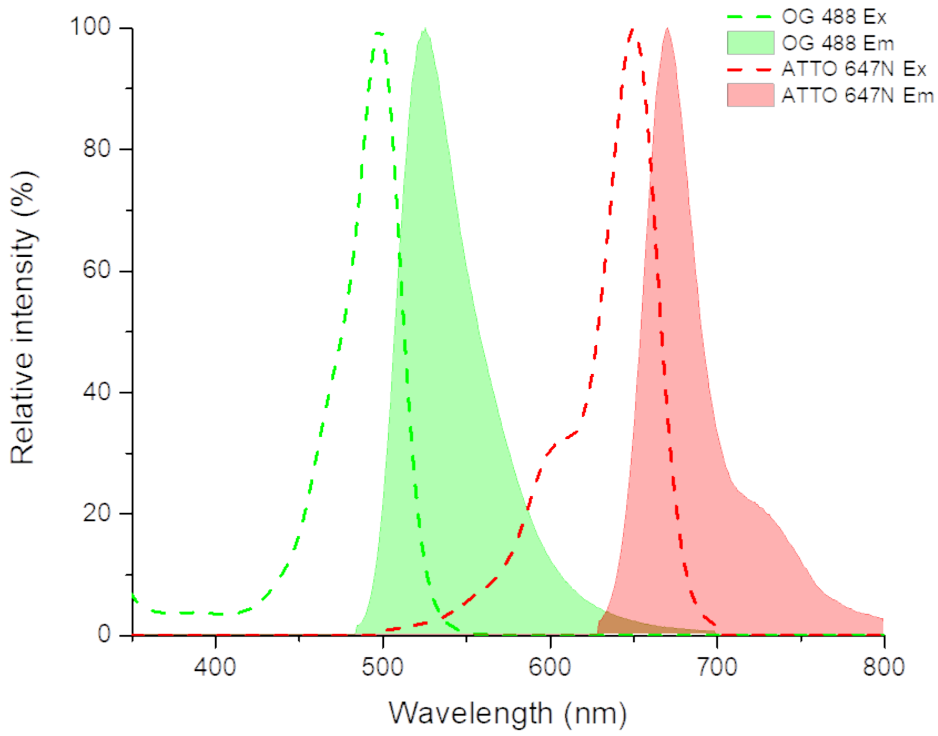

Figure S1. Absorption and emission spectra of Oregon green 488 and ATTO 647N. (Data obtained by Fluorescence spectraviewer).

S1. Fluorescent emission and excitation spectra of Oregon Green 488 and ATTO 647N.

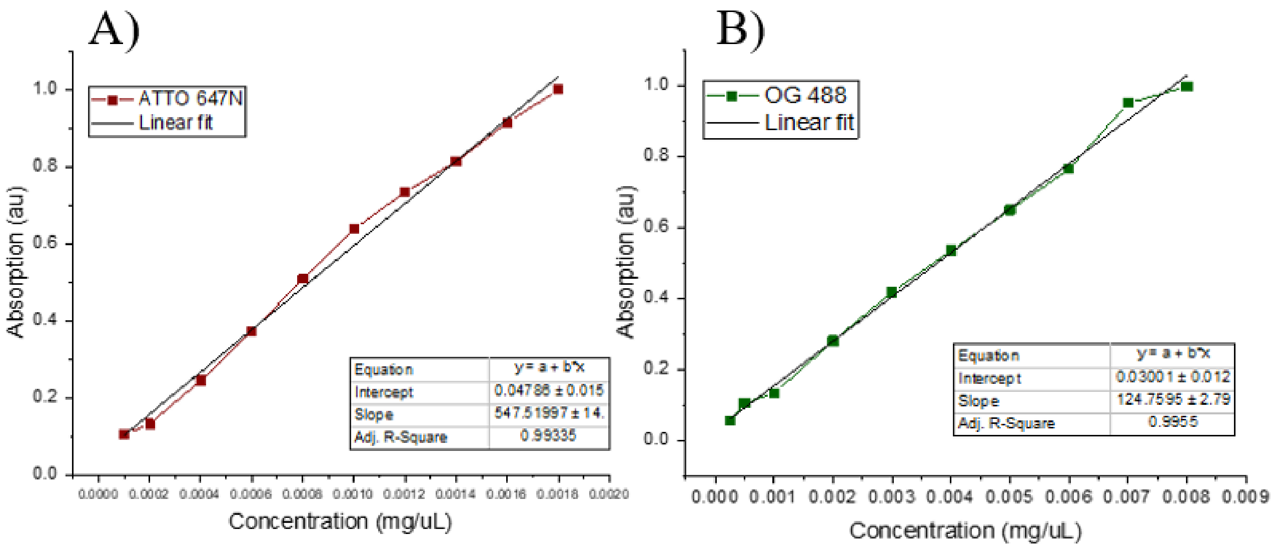

Figure S2. Different amounts of ATTO 647N (A) and GO 488 (B) were diluted in ultrapure milli-Q water. Every dilution was measured by UV-vis spectroscopy using a Nanodrop 2000c device (at 650 nm and 493 nm for ATTO 647N and Oregon green 488 respectively). Linear fitting of data was analyse using OriginPro software.

S3. Analysis of the influence of the encapsulation of the dyes in the fluorescent spectrum.

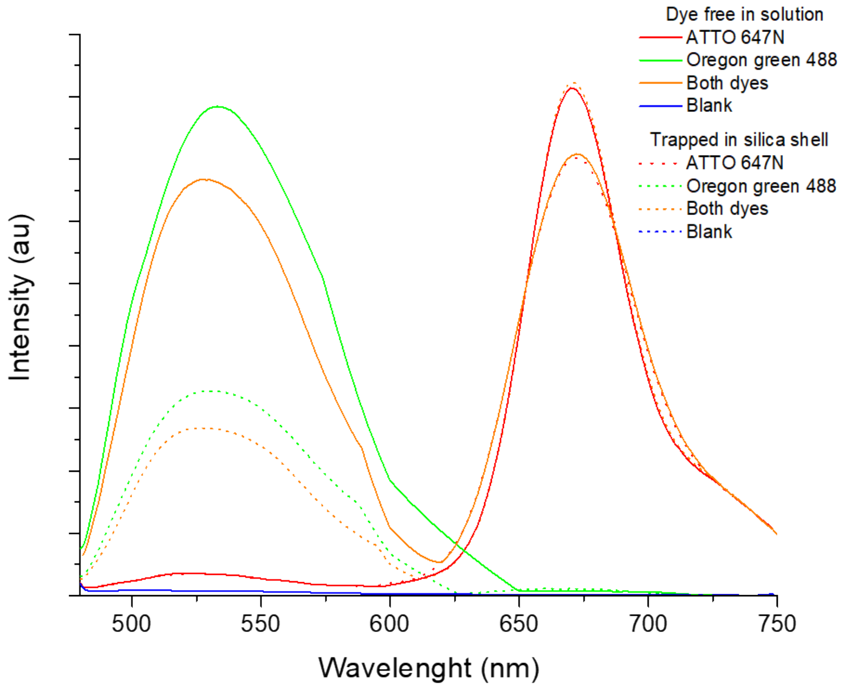

Figure S3. Fluorescent spectra of two commercial dyes in solution (solid line) and trapped in their silica shell (dot line): -ATTO 647N, -Oregon green 488, -both dyes present and -blank. For the encapsulated dyes, all the nanosystems were synthesized under the same protocol described, varying only the presence of dyes. For the dyes free in solution, both dyes were fixed at the same concentration. Fluorescent spectra and intensities were compared both individually and both present in the same condition (solution or silica encapsulation). The measurements were performed by a TECAN spectrophotometer using a 96-well plate. Ethanol was used as blank.

S4. Spectrophotometric determination of biotin using HABA/Avidin reagent (SIGMA H-2153).

The protocol is based on the HABA dye binding to avidin and the ability of biotin to displace the dye in stoichiometric proportions. This displacement of dye is accompanied by a change in absorbance at 500 nm.

Protocol

1. Reconstitution of vial with 10 mL deionized water (pH 7.3).
2. In 1 mL cuvette add 900 μL HABA/Avidin reagent previously reconstituted.
3. Read absorbance at 500 nm ($A_{HABA/Avidin}$).
4. Add 100 μL of sample and read absorbance at 500 nm (A_{sample}).
5. Prepare a blank sample diluting 100 μL of sample in 900 μL of water and read at 500 nm (A_{blank}).

Calculations

$$\Delta A_{500} = (0.9 A_{HABA\,/\,Avidin}) + A_{blank} - A_{sample}$$

Where 0.9 corresponds to the dilution factor of HABA/avidin reagent

$$\text{Biotin concentration} \left[\frac{\mu\text{mol}}{\text{mL}}\right] = \left(\frac{\Delta A_{500}}{34}\right) * 10$$

Where 34 and 10 correspond to the extinction coefficient at 500 nm and the dilution factor of the sample into the cuvette, respectively.

N. M. Green, *Biochemical Journa*l, 1965, **94** 23.
N. M. Green, *Methods Enzymol,* 1970. **18**, 418.

S5. Fourier-transform infrared (FT-IR) spectra of biotinylated DC 50 nanosystem.

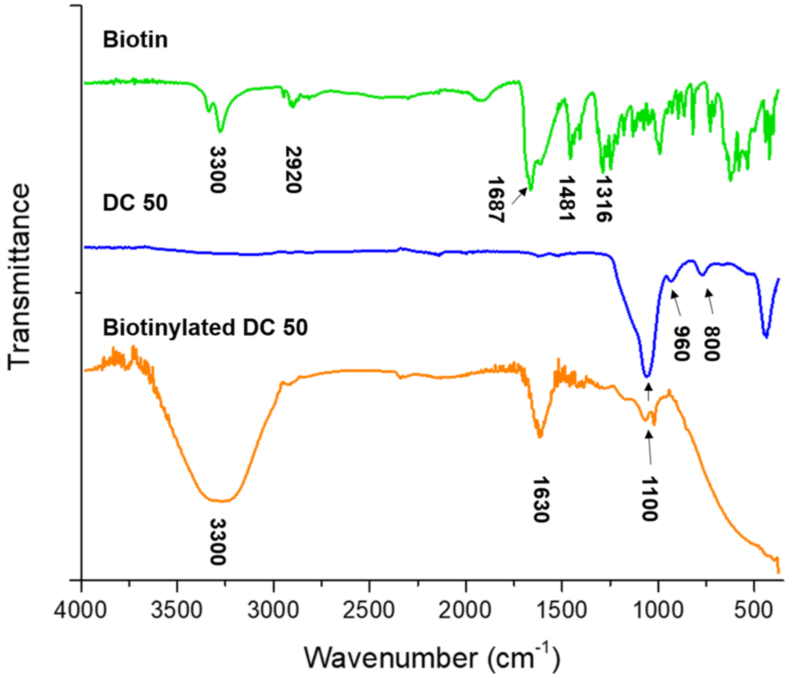

Figure S5. FT-IR spectra of biotin (green line), DC 50 (blue line) and biotinylated DC 50 (orange line). The transmittance spectrums from 350 to 4000 cm^{-1} are shown. The spectrum of biotinylated DC 50 is compared with biotin solution using the same biotin concentration found in the DC 50 solution. Signals are observed close to 3300 cm^{-1}, 1316 cm^{-1}, 1481 cm^{-1} and 1639 cm^{-1} corresponding to skeletal vibrations of biotin C–C, C=O stretch and C–O deformation band respectively. For DC 50 and biotinylated DC 50 (blue and orange lines respectively) are observed three typical signals close to 800 cm^{-1}, 960 cm^{-1} and 1100 cm^{-1} related to the Si-O-Si bending vibrations, Si-OH stretching vibration and Si-O groups.

N. A. Lapin, Y. J. Chabal, *J Phys Chem B*, *2009*, ***113***, 8776.

V. Balan, I. Petrache, I. M. Popa, M. Butnaru, E. Barbu, J. Tsibouklis, L. Verestiuc, *Journal of Nanoparticle Research* 2012, **14.** 730.

M. Emami, A. Teimouri, A. Chermahini, Spectrochimica acta. Part A, 2008, **71,** 1516.

T. Tran, T. Pham, P. Le, T. Nguyen, V. Tran, *Adv. Nat. Sci*. 2013, **4**, 045007.

T. Oh, *Korean Phys. Soc.* 2010, **56**. 1150.

S6. pH response of encapsulated fluorophores in SiO_2 NPs

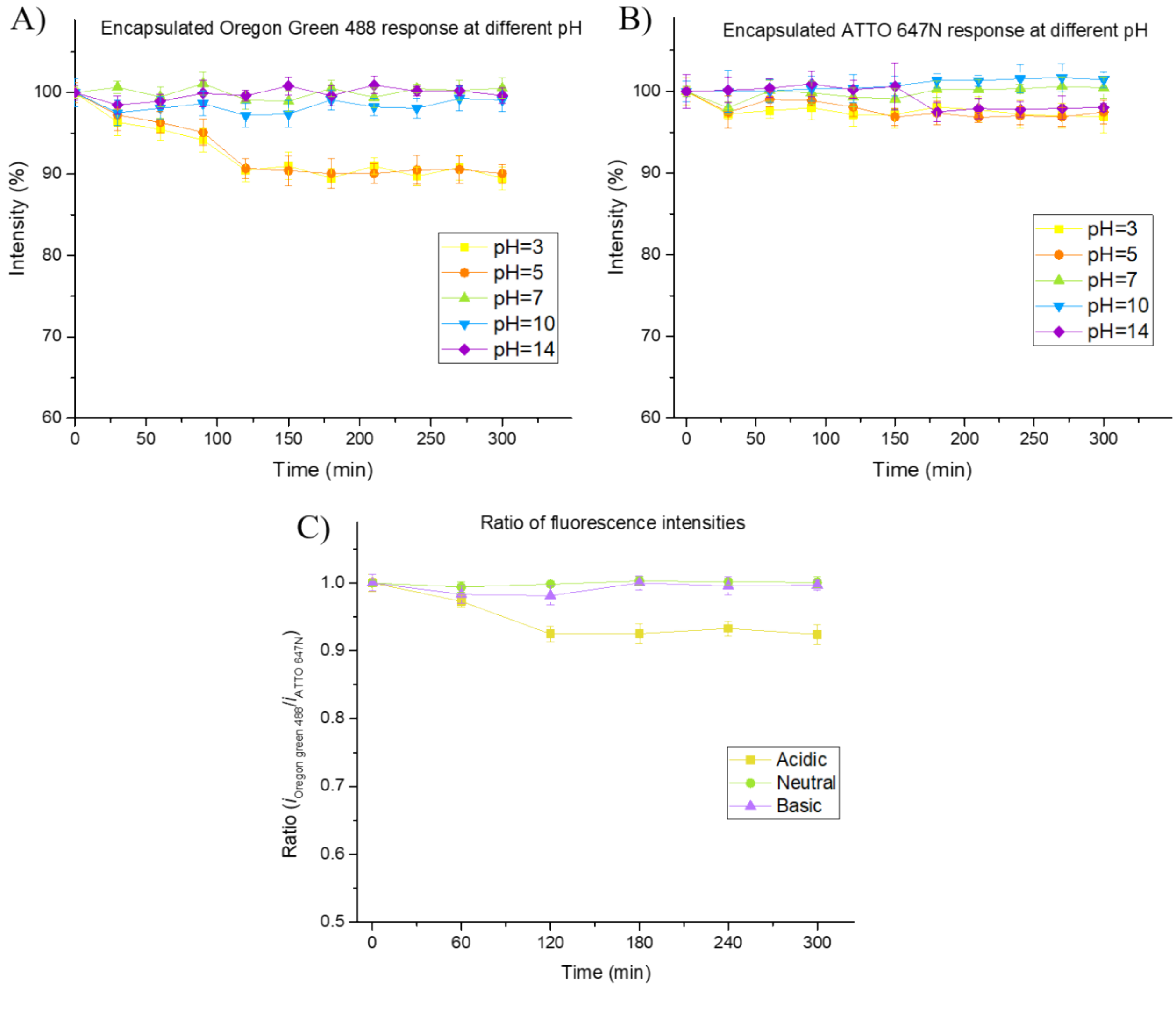

Figure S6. pH fluorescence response of A) encapsulated OG 488 in SiO_2 NPs (λ_{ex}= 450 nm), B) encapsulated ATTO 647N in SiO_2 NPs (λ_{ex}= 650) and C) OG 488 versus ATTO 647N intensity ratio at different pH by time. To study the behaviour of both encapsulated dyes in SiO2 NPs at different pH, hydrochloric acid (HCl) and sodium hydroxide (NaOH) were used to set the pH at 3, 5, 10 and 14. The biotinylated DC 50 nanosystem was used for the measurements comparing the fluorescent intensities by a spectrophotometer (TECAN 200 PRO NanoQuant dispositive). The fluorescent spectrum was obtained every 30 minutes for a period of 5 hours using an excitation wavelength of 450 nm and 650 nm for OG488 and ATTO 647N respectively. Data show that the bicolor nanoparticles ratiometrically respond to medium acidification.
